# Construction of a nomogram to predict the probability of new vertebral compression fractures after vertebral augmentation of osteoporotic vertebral compression fractures: a retrospective study

**DOI:** 10.3389/fmed.2024.1369984

**Published:** 2024-04-23

**Authors:** Yan Gao, Jianhu Zheng, Kang Yao, Weiguo Wang, Guoqing Tan, Jian Xin, Nianhu Li, Yungang Chen

**Affiliations:** ^1^Shandong University of Traditional Chinese Medicine, Jinan, China; ^2^The First Clinical Medical College of Shandong University of Traditional Chinese Medicine, Jinan, China; ^3^Affiliated Hospital of Shandong University of Traditional Chinese Medicine, Jinan, China

**Keywords:** osteoporotic vertebral compression fractures, new vertebral compression fractures, percutaneous kyphoplasty, nomogram, clinical prediction model

## Abstract

**Objective:**

This study aimed to develop and validate a new nomogram model that can predict new vertebral fractures after surgery for osteoporotic compression fractures to optimize surgical plans and reduce the incidence of new vertebral compression fractures.

**Methods:**

420 patients with osteoporotic vertebral compression fractures were randomly sampled using a computer at a fixed ratio; 80% of the patients were assigned to the training set, while the remaining 20% were assigned to the validation set. The least absolute shrinkage and selection operator (LASSO) regression method was applied to screen the factors influencing refracture and construct a predictive model using multivariate logistic regression analysis.

**Results:**

The results of the multivariate logistic regression analysis showed a significant correlation between bone cement leakage, poor cement dispersion, the presence of fractures in the endplate, and refractures. The receiver operating characteristic curve (ROC) results showed that the area under the ROC curve (AUC) of the training set was 0.974 and the AUC of the validation set was 0.965, which proves that this prediction model has a good predictive ability. The brier score for the training set and validation set are 0.043 and 0.070, respectively, indicating that the model has high accuracy. Moreover, the calibration curve showed a good fit with minimal deviation, demonstrating the model’s high discriminant ability and excellent fit. The decision curve indicated that the nomogram had positive predictive ability, indicating its potential as a practical clinical tool.

**Conclusion:**

Cement leakage, poor cement dispersion, and presence of fractures in the endplate are selected through LASSO and multivariate logistic regressions and included in the model development to establish a nomogram. This simple prediction model can support medical decision-making and maybe feasible for clinical practice.

## Introduction

With the aggravation of the aging population, the incidence rate of osteoporosis in the older population has shown a significant upward trend, accompanied by severe bone loss. Presently, osteoporosis has been listed by the World Health Organization as one of the ten most serious diseases worldwide (1). Osteoporotic vertebral compression fractures (OVCF) are one of the most common complications of osteoporosis (2). Approximately 20% of older people aged >70 years and 16% of postmenopausal women experience OVCF. Moreover, OVCF usually adversely affects the quality of life, physical function, mental health, and quality of life of patients, which are related to the degree of kyphotic deformity and pain caused by fractures (3). Percutaneous kyphoplasty (PKP) is widely used in clinical treatment because of its technical safety and effective pain relief. However, some studies have reported the risk of bone cement leakage and new vertebral compression fractures (NVCF) after PKP surgery (4, 5).

NVCF is a common complication of OVCF surgery that further reduces the quality of life of patients and places a huge economic burden on society. Patients with a first occurrence of OVCF have an increased risk of experiencing NVCF (6), which may be related to the natural progression of osteoporosis (7). Cheng et al. (8) showed that, of 247 patients who underwent vertebral augmentation surgery, 23 (9.3%) developed NVCF. Seo et al. (9) reported that the stiffness and strength of the vertebral body were strengthened after injecting bone cement into the fractured vertebral body, thereby increasing the risk of other vertebral fractures. However, Yi et al. (10) found no significant difference in the incidence of adjacent vertebral fractures after surgery between patients who received bone cement injections and those who received conservative treatment. In addition, there are many studies on OVCF and multiple risk factors that easily cause NVCF, such as bone cement dispersion, bone cement leakage, anti-osteoporosis treatment, bone mineral density (BMD), and fracture location (11–13).

Currently, the risk of postoperative new vertebral compression fractures (NVCF) in patients undergoing percutaneous kyphoplasty (PKP) has been widely recognized and cautioned against. Previous studies have identified independent risk factors associated with it (14, 15). However, few studies have utilized visual mathematical models to present the data more clearly and intuitively. In recent years, an increasing number of scholars (12, 16–19) have conducted related research and established various nomograms. These studies have included sample sizes of over 300 individuals and follow-ups of 2 years or more, demonstrating good sensitivity and specificity. However, most studies have been based on single surgical teams in individual medical centers, with few multicenter studies and external validations. Additionally, these models collect different types and quantities of variables, leading to differences in the predictive factors analyzed. A literature review reveals that the observed indicators in these studies include general information, surgical factors, imaging findings, etc., but laboratory tests have not been included in the observed indicators. It is undeniable that biomarkers such as bone-specific alkaline phosphatase, vitamin D, parathyroid hormone, etc., are closely related to the pathogenesis of osteoporosis. Therefore, these results may not fully reflect reality and may hinder clinical decision-making regarding post-PKP preventive measures. In this study, we aim to develop a Nomogram model by incorporating more comprehensive observed indicators to predict patient prognosis and assess the probability of postoperative NVCF occurrence. By applying this model, surgeons can better prevent the recurrence of vertebral compression fractures after PKP and avoid unnecessary waste of medical resources. Current research is limited to the development and validation of the nomogram and has not investigated its clinical application. Therefore, we have designed an operational flowchart for clinical doctors, showcasing the data generated from the hospital’s EMR system and manually assessed imaging information, which is presented in the Appendix.

## Methods

### Clinical data and selection criteria

This study retrospectively analyzed 698 patients with OVCFs who underwent PKP surgery at the Affiliated Hospital of Shandong University of Traditional Chinese Medicine between January 2016 and January 2021. The follow-up period for all patients was 2 years. This research was approved by the Institutional Research Ethics Committee of the Shandong University of Traditional Chinese Medicine Affiliated Hospital. Written informed consent was obtained from all patients who underwent the PVP procedure interpretation and clinical data processing. This study met the specifications for the Transparent Reporting of a Multivariable Prediction Model for Individual Prognosis or Diagnosis (TRIPOD) (20).

**Figure 1:**
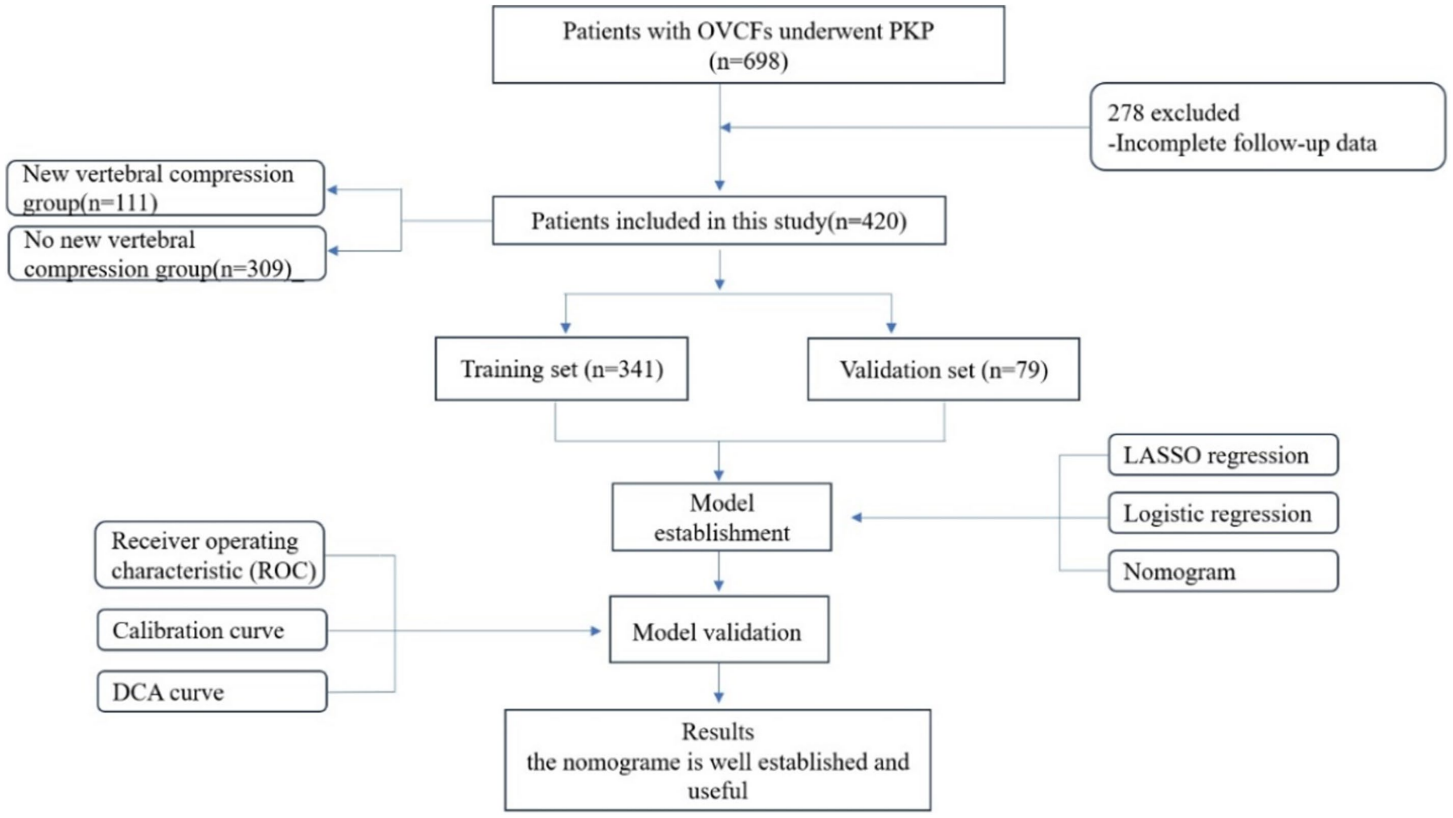
Flowchart of study participants.

The inclusion criteria were as follows: 1. Primary osteoporosis with a bone density that met the World Health Organization diagnostic criteria for osteoporosis; 2. Availability of complete preoperative basic data, imaging data, laboratory examinations, and re-examination at the designated time after surgery; 3. Patients who had undergone PKP surgery; 4. Presence of obvious low back pain (visual analog score > 6 points), limited physical activity, especially when rolling over or waking up; 5. Patients with magnetic resonance imaging (MRI) that had revealed significant signal changes in the thoracic and lumbar vertebrae, with high-intensity T2 and low-intensity T1 signals appearing in the fractured vertebral body or whole-body bone scans showing active bone metabolism; 6. Intact posterior wall of the vertebral body that the fracture did not affect, and the vertebral canal was not invaded; and 7. Absence of heart, liver, or other organ failures before surgery. The exclusion criteria were as follows: 1. OVCF caused by a tumor, infection, or tuberculosis; 2. patients who had coagulation dysfunction combined with systemic diseases and could not tolerate surgery; 3. preoperative systemic or local infections; 4. the presence of spinal cord compressions and obvious neurological symptoms, such as lower limb numbness and muscle atrophy; 5. acceptance of posterior pedicle screw fixation and bone graft fusion; and 6. presence of fractures in the posterior wall of the vertebral body, which can lead to mechanical instability (Figure 1).

### Percutaneous kyphoplasty

In this study, all patients underwent PKP using a unilateral pedicle approach. The amount of bone cement injected preoperatively was estimated based on the size, degree of compression, and completeness of the vertebral body. All patients were placed in the prone position with support pads placed below the chest and pelvis to avoid abdominal pressure. Routine preoperative towel disinfection and full monitoring of vital signs were conducted. The fractured vertebral was localized using puncture needles under the guidance of C-arm fluoroscopy. The puncture needle was placed one-third anterior to the fractured vertebral body through the pedicle under fluoroscopy lateral radiographs. Then take out the puncture needle was removed, and the balloon was inflated to restore the vertebral height in the PKP. The “toothpaste-like” polymethylmethacrylate (PMMA) was instilled, filling the fractured bone. The whole process was slowly completed under fluoroscopy to avoid bone cement leakage. All the procedures were completed successfully by two experienced orthopedic surgeons.

### New OVCF identification criteria

The main diagnostic criteria for NVCF after PKP were as follows: 1. After receiving PKP treatment for the initial fracture, lower back pain recurrence within 2 years of follow-up, accompanied by limited mobility and increased pain when changing positions, such as waking up and turning over. Physical examination revealed local tenderness and percussion pain without symptoms of spinal cord or nerve root compression. 2. Compared to the radiographic appearance of the first fracture, there was a new vertebral wedge transformation, and MRI showed a low signal on T1-weighted images and a high signal on T2-weighted images. MRI also was used to exclude other spinal diseases, including infections and malignant tumors, thus helping us exclude patients who did not meet the NVCF standards.

### Statistical analysis

#### Selection criteria for predictor variables

We analyzed and reviewed relevant literature published to date, summarizing and analyzing patient baseline data, laboratory tests, imaging examinations, surgeries, and other aspects, and collected risk factors related to NVCF. Baseline features included age, sex, BMI (weight in kilograms divided by height in meters), BMD, time from injury to surgical treatment, length of hospital stay, fracture site, presence of multiple vertebral fractures, history of steroid use, and regular anti-osteoporosis treatment after the first surgery. Imaging examinations included local vertebral height recovery, vertebral height recovery rate factors, such as changes in the Cobb angle, presence of endplate fractures, leakage of bone cement (defined as bone cement entering the intervertebral space beyond the upper/lower vertebral lamina), and good dispersion of laboratory indicators, such as osteocalcin, vitamin D, and parathyroid hormone. Surgical factors included the amount of bone cement injected and the number of vertebral bodies undergoing surgery. Baseline characteristics and laboratory indicators were extracted using ETLCloud software. Surgical factors were obtained by reviewing patient surgical records. Imaging information was evaluated by two spine surgeons (with 3 years and 7 years of clinical experience, respectively) using imaging system software to record imaging measurement results. In case of disagreement, a group discussion was held with another surgeon (with 20 years of clinical experience) to reach a consensus and record it.

#### Statistical methods and software

In this study, STATA 17.0 for Windows (StataCorp, Texas, United States) and Rversion 4.1.3 (RFoundation for Statistical Computing) were used for statistical analysis. Continuous variables were statistically analyzed using the Mann–Whitney U test and expressed as medians (quartiles). Categorical variables were statistically analyzed using the chi-square or Fisher exact tests and reported as the number of cases (percentage). Using a computer to conduct random sampling at a fixed proportion, 80% of the patients were assigned to the training set, and the remaining 20% were assigned to the validation set. The LASSO regression method was used to reduce the data dimensions, avoid overfitting and multicollinearity, and screen the factors influencing refracture. Use 10x cross-validation to determine the optimal value through 1x standard error λ. Based on the characteristics of LASSO regression screening, a predictive model was established using multivariate logistic regression analysis, and the nomograms and calibration plots were established in the training set using R software and the “rms” software package. Using Stata software to draw the ROC curve, the larger the AUC, the stronger the predictive ability of the model. The Stata software was used to draw decision curves, quantitatively verify the net benefits under different threshold probabilities in the dataset, and conduct decision curve analysis to determine the clinical practicality of the column chart. All statistical tests were bilateral, and a *p*-value <0.05 was considered significant.

#### Ethics approval and consent to participate

The study was conducted by the Declaration of Helsinki. The Ethics Committee of the Affiliated Hospital of Shandong University of Traditional Chinese Medicine approved the study. Because it was a retrospective study, written informed consent was waived.

## Results

### Patients’ baseline characteristics

Overall, 420 patients met the inclusion criteria; 111 patients were newly diagnosed with fractures after surgery, whereas 309 patients had no new fractures. In the group without new fractures, the number of female patients (279 cases, 90.3%) was significantly higher than that of male patients (30 cases, 9.7%), presenting the same results in the new fracture group, indicating that there was no statistically significant difference in sex between the two groups. In addition, there were no significant differences (*p* > 0.05) between the two groups in terms of age, menopausal age, body mass index (BMI), BMD, time from injury to surgical treatment, length of hospital stay, fracture site, presence of multiple vertebral fractures, history of steroid use, vitamin D levels, parathyroid hormone levels, Injection volume, vertebral height recovery rate, or Cobb angle recovery. There was a significant difference in osteocalcin levels between the two groups (*p* < 0.01), with lower levels of osteocalcin in the blood of patients in the refracture group. More patients in the newly diagnosed fracture group experienced preoperative vertebral endplate fractures and intraoperative bone-cement leakage with poor bone-cement dispersion (*p* < 0.01); Table 1 presents the detailed results.

**Table 1:** Baseline characteristics of patients with no new vertebral compression fractures and new vertebral compression fractures.

Variables	No new vertebral compression (*n* = 309)	New vertebral compression (*n* = 111)	*p*-value
Gender (*n*, %)			
Male	30 (9.7%)	15 (13.5%)	0.28
Female	279 (90.3%)	96 (86.5%)	
Age [years]			
≤70	138 (44.7%)	42 (37.8%)	0.35
80>age>70	115 (37.2%)	43 (38.7%)	
≥80	56 (18.1%)	26 (23.4%)	
Menopausal age [years]			
Male	30 (9.7%)	15 (13.5%)	0.54
≤47	70 (22.7%)	20 (18.0%)	
52>age>47	172 (55.7%)	61 (55.0%)	
≥53	37 (12.0%)	15 (13.5%)	
Multiple vertebral fracture			
No	251 (81.2%)	80 (72.1%)	0.06
Yes	58 (18.8%)	31 (27.9%)	
Fracture site			
Over T10	21 (6.8%)	10 (9.0%)	0.07
T10–L2	211 (68.3%)	69 (62.2%)	
L3–L5	45 (14.6%)	11 (9.9%)	
Else	32 (10.4%)	21 (18.9%)	
Steroid use			
No	300 (97.1%)	108 (97.3%)	1.00
Yes	9 (2.9%)	3 (2.7%)	
Anti-osteoporosis therapy			
No	136 (44.0%)	67 (60.4%)	<0.01
Yes	173 (56.0%)	44 (39.6%)	
Leakage			
No	286 (92.6%)	19 (17.1%)	<0.01
Yes	23 (7.4%)	92 (82.9%)	
Diffusion of bone cement			
No	28 (9.1%)	93 (83.8%)	<0.01
Yes	281 (90.9%)	18 (16.2%)	
Endplate fracture			
No	289 (93.5%)	12 (10.8%)	<0.01
Yes	20 (6.5%)	99 (89.2%)	
Hospitalization to surgery (days) (median, (IQR))	3.0 (2.0, 4.0)	3.0 (2.0, 4.0)	0.30
Injection volume (ml) (median, (IQR))	5.0 (4.0, 5.0)	4.5 (3.5, 5.0)	0.11
BMI (kg/m^2^) (median, (IQR))	23.4 (21.5, 25.2)	22.9 (20.5, 26.4)	0.78
BMD (median, (IQR))	0.6 (0.6, 0.7)	0.6 (0.6, 0.7)	0.06
Parathyroid hormone (median, (IQR))	40.7 (30.7 53.5)	40.2 (31.9,51.9)	0.72
Vitamin D (median, (IQR))	15.6 (11.5, 22.7)	16.3 (11.9, 23.1)	0.60
Osteocalcin (median, (IQR))	18.5 (14.4,26.1)	15.9 (12.0, 23.5)	<0.01
Operation time after injury (days) (median, (IQR))	9.0 (4.0, 17.0)	10.0 (6.0, 21.0)	0.09
Fractured vertebral bodies (numbers) (median, (IQR))	1.0 (1.0, 1.0)	1.0 (1.0, 2.0)	0.04
Hight recover (mm) (median, (IQR))	9.4 (6.3,11.9)	9.1 (6.5,12.1)	0.90
High recovery rate (median, (IQR))	0.7 (0.4, 0.9)	0.7 (0.4, 1.0)	0.12
Cobb angle recover(°) (median, (IQR))	9.7 (6.9,13.2)	11.3 (6.7,15.7)	0.07

### Least absolute shrinkage and selection operator regression analysis results

**Figure 2:**
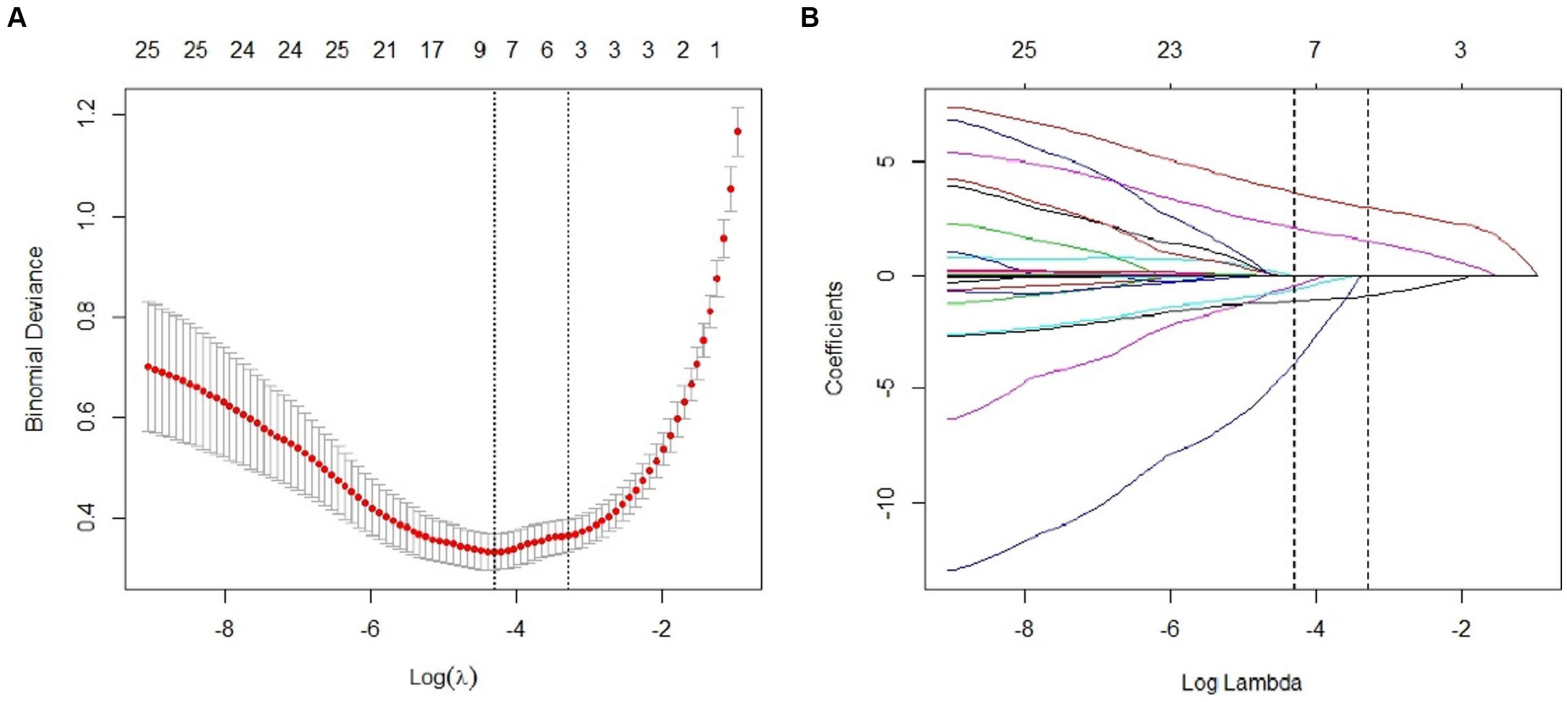
Predictor selection using the least absolute shrinkage and selection operator (LASSO) logistic regression model. **(A)** Dotted vertical lines were drawn at the optimal values by using the minimum criteria and the 1 standard error of the minimum criteria (the 1-SE criteria). **(B)** Lasso coefficient profiles of the 22 clinical features. The dotted vertical line was plotted at the value selected using 10-fold cross-validation in **(A)**, for which the optimal λ resulted in 3 non-zero coefficients.

Using LASSO regression analysis for feature variable screening, variable shrinkage coefficient graphs and cross-validation curve graphs were drawn. According to the results of cross-validation, when the mean square error of the distance was twice the standard error (λ 1se) and λ1se = 0.04578116, the optimal model was obtained, and three non-zero coefficient research variables were selected, namely bone cement leakage, poor bone cement dispersion, and the presence of endplate fractures. Figure 2 shows the detailed results.

### Establishing a multivariate logistic regression analysis model

A multivariate logistic regression analysis model was constructed using the three characteristic variables identified by LASSO regression, including cement leakage, poor cement dispersion, and the presence of fractures in the endplate, as independent variables and the occurrence of refractures as dependent variables. The results showed that the leakage of bone cement, poor dispersion of bone cement, and presence of fractures and refractures in the endplate were all significant (*p* < 0.01); Table 2 shows the detailed results.

**Table 2:** Multivariate logistic regression in the training set.

Variable	Multivariate logistic analysis		
	β	Odds ratio (95% CI)	*p*-value
Leakage	2.411	11.148(3.514–35.366)	0.014
Diffusion of bone cement	−1.512	0.221 (0.659–0.738)	<0.001
Endplate fracture	3.772	43.449 (13.233–142.657)	<0.001

### Development and validation of nomogram diagrams

**Figure 3:**
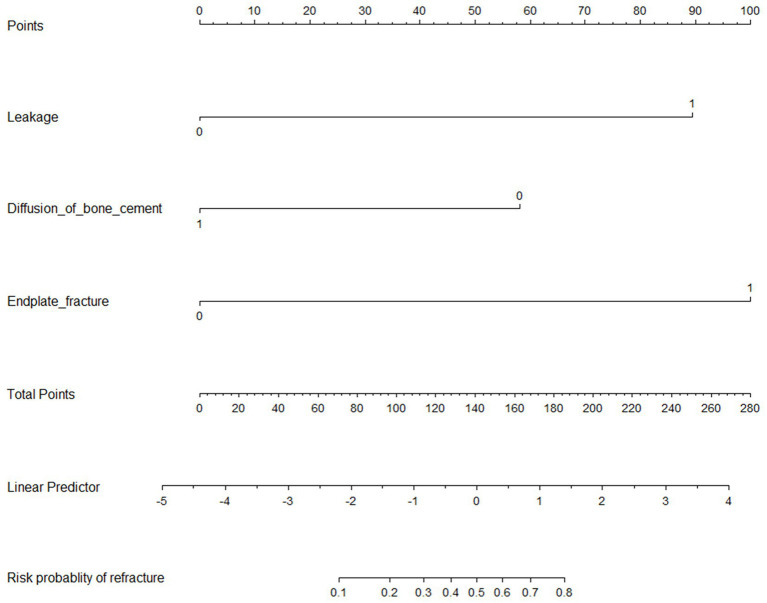
Nomogram to predict the probability of new vertebral compression fractures after vertebral augmentation of osteoporotic vertebral compression fractures.

**Figure 4:**
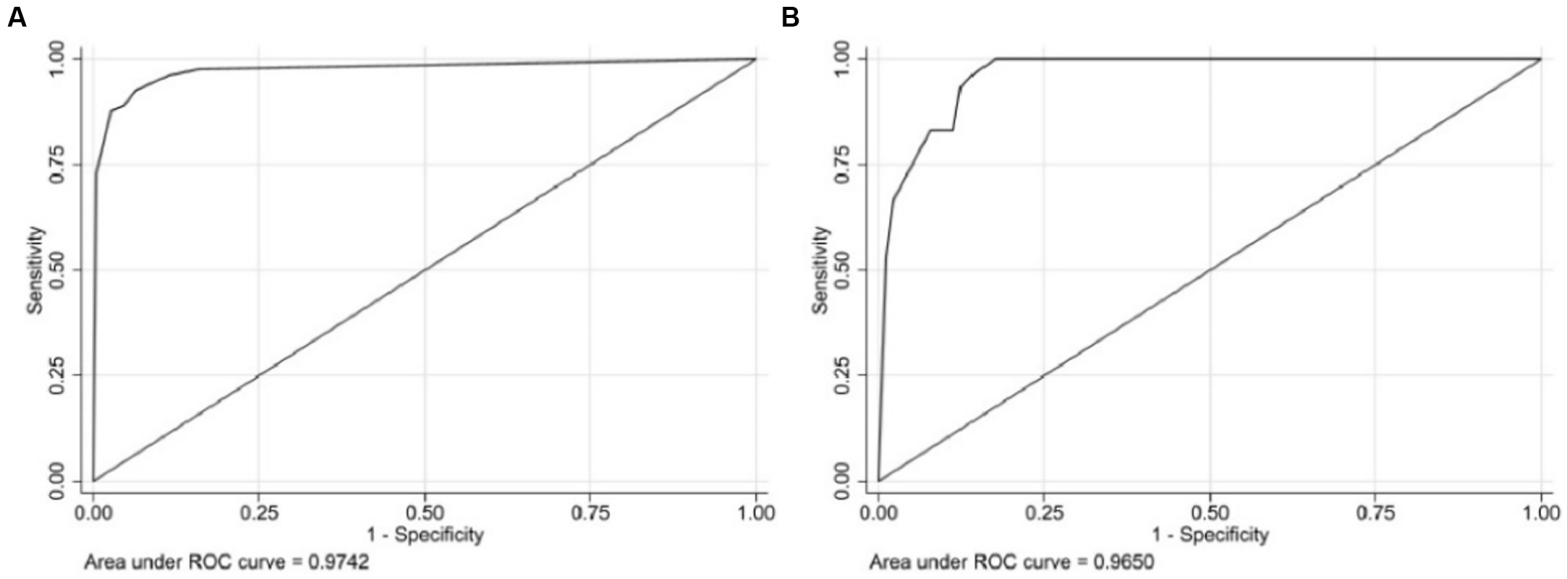
The receiver operating characteristic curve (ROC) of the nomogram, with an area under the ROC curve (AUC) of 0.9742 in the training cohort **(A)** and an AUC of 0.9650 in the validation cohort **(B)**.

**Figure 5:**
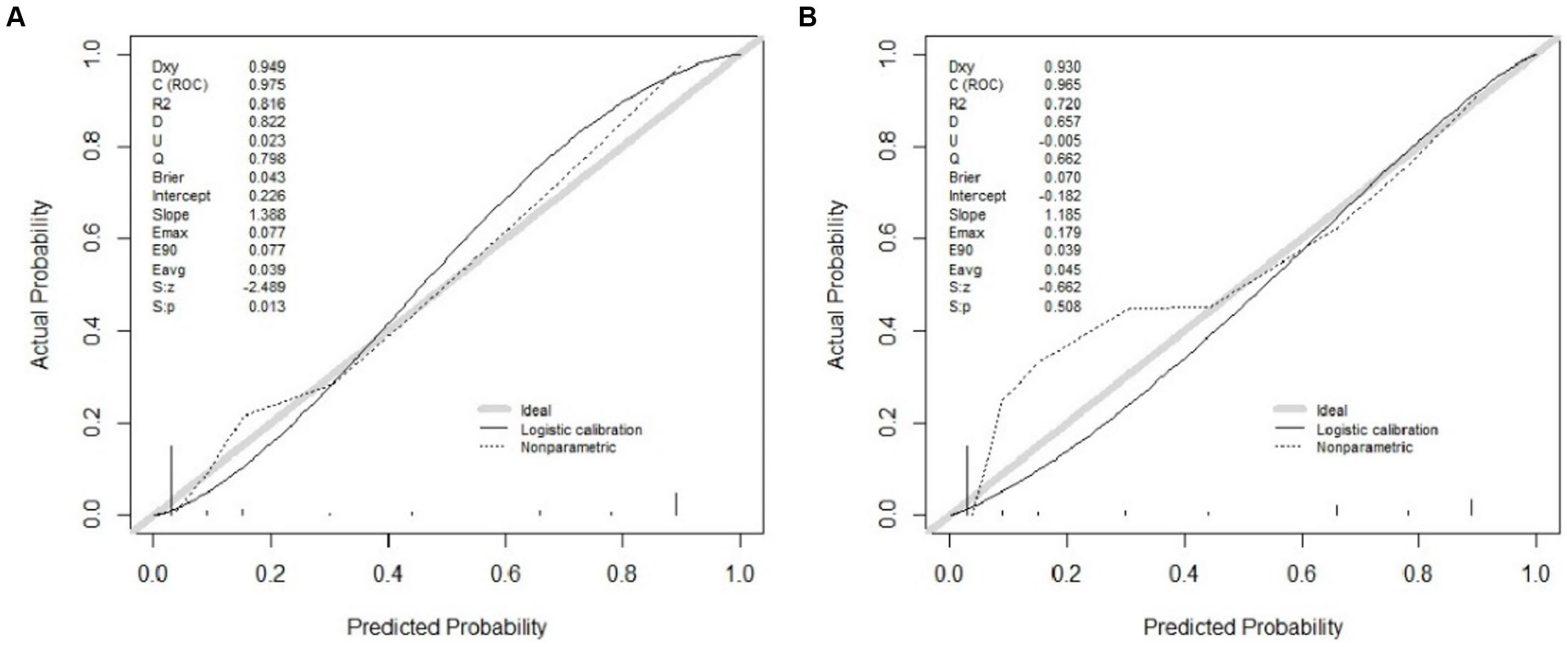
Calibration curves of the nomogram in the training **(A)** and validation **(B)** cohort. The x-axis represents the predicted probability calculated by the nomogram, and the y-axis is the observed actual probability of the new vertebral compression fracture. The clinodiagonal represents a perfect prediction by an ideal model. The black solid line represents the performance of the model after calibration.

Three independent predictive factors were selected through LASSO and multivariate logistic regressions and included in the model development to establish a nomogram. Among these are three predictive factors as follows: cement leakage, poor cement dispersion, and presence of fractures in the endplate (Figure 3). Each prediction factor was located on the relevant axis, and a straight line was drawn based on the prediction factor on the vertex axis to obtain a point. The scores obtained for each predictor were summed to obtain the total score. The final total score was placed on the total score axis, and a straight line was drawn to predict the probability of further fractures. To further determine the accuracy of the nomogram plot, we validated it using the receiver operating characteristic and calibration curves. The ROC curve results showed that the area under the ROC curve (AUC) of the training set was 0.974 and the AUC of the validation set was 0.965 (Figure 4), which proves that this prediction model has a good predictive ability. The calibration curve of the model fits well with the curve after correcting for bias, indicating that the prediction model has a high discrimination ability and good fitting performance (Figure 5).

### DCA curve analysis

**Figure 6:**
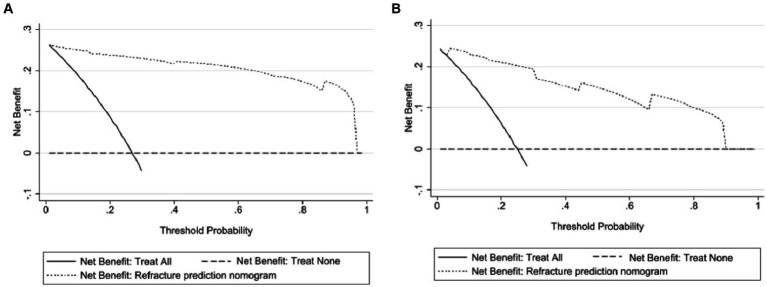
Clinical decision curves for the nomogram model. **(A)** Indicates the decision curve for the training set, and **(B)** indicates the decision curve for the validation set. The x-axis shows the threshold probability, and the y-axis shows the net beneft. The horizontal line indicates no patients develop new vertebral compression fracture, and the black oblique line indicates patients develop new vertebral compression fracture. The dashed line in the upper right corner represents the new vertebral compression fracture risk nomogram. In DCA, the nomogram shows a more net benefit than full or no treatment across a threshold probability range.

As shown in Figure 6, within a threshold range of approximately 0.1 to 0.9, using a predictive model to predict the risk of refracture within 2 years after surgery for osteoporotic compression fractures was more advantageous than implementing intervention plans for all patients. The net benefit of the predictive model was significantly higher than that of full intervention or non-intervention for patients.

## Discussion

With the aging population, osteoporosis has become a common disease that threatens the health of middle-aged and older individuals. OVCF is one of the most common brittle fractures, with a 30–50% prevalence rate in people over 50 years old (21). PKP, one of the best options for treating OVCF, can effectively alleviate pain symptoms (22) and many postoperative complications. Related studies have shown that the probability of recurrent vertebral fractures in patients with OVCFs undergoing PKP surgery can be as high as 34.8% (13, 23). According to the published literature, factors such as fracture site, cement leakage, advanced age, sagittal biomechanical imbalance, and low bone density are independent risk factors for NVCF. In this study, we used LASSO and multivariate logistic regression to screen for three independent predictive factors: cement leakage, cement dispersion, and the presence or absence of endplate fractures. We developed a nomogram model that has the advantages of simplicity, convenience, and reliable results. This study also used a validation set to validate the established model. The calibration curve showed that the model had high applicability, reflecting its clinical application value. This model can be used to assist spinal surgeons in assessing the risk of recurrent fractures in patients with OVCF after PKP surgery and to develop personalized prevention and treatment strategies for such high-risk patients.

Bone cement leakage is a common complication after PKP surgery, and the literature has reported that it is an important factor influencing NVCF (24, 25). In a study on bone cement leakage, approximately 14.7% of 102 patients with PKP experienced bone cement leakage (26). Dai et al. conducted a meta-analysis of 23 articles, including 9,372 patients with OVCF, of which 1,255 (13.39%) had postoperative refractures. The results showed that bone cement leakage (OR = 2.05, 95% CI 1.40–3.00, *p* < 0.001) increased the risk of postoperative refractures and was a closely related risk factor, consistent with the results of this study. Bone cement leakage has always been a research hotspot among the postoperative complications of minimally invasive surgery. Bone cement can leak into different body parts, and common clinical sites include the intervertebral disc, soft tissue near the vertebral canal, venous plexus of the vertebral canal, and epidural space. Some studies have shown that leakage of bone cement into the intervertebral disc is a critical factor causing postoperative fractures (odds ratio = 4.633) (27). The intervertebral disc is a crucial buffering device in the spinal mechanical system. Infiltration of bone cement into the intervertebral disc tissue can exacerbate the degeneration of the intervertebral disc, leading to an uneven distribution of stress within the intervertebral disc, thereby weakening the buffering capacity of the intervertebral disc and the strength of adjacent vertebral endplates (28, 29), increasing the risk of other vertebral fractures. In 2008, through a retrospective analysis of 508 patients who underwent percutaneous vertebroplasty (PVP) surgery, Ahn et al. (30) believed that fractures in adjacent vertebral bodies were caused by a significant difference in strength between adjacent vertebral bodies after filling with bone cement and referred to this phenomenon as direct pillar effect. In addition, nonadjacent vertebral fractures are defined as a dynamic hammer effect, which may be due to the low strength and poor mobility of adjacent vertebral bodies, thereby affecting the distal vertebral body and causing fractures. Therefore, if bone cement leakage occurs during surgery, patients should be promptly informed of the high likelihood of recurrent fractures and other risks after surgery (31). Based on previous reports and the results of this study, we believe that it is necessary to carefully observe the morphology of the fractured vertebral body, the position of the fracture line, and the approach and angle of the puncture needle before surgery to avoid leakage of bone cement.

In this study, we defined the dispersion mode of bone cement within the vertebral body and believed that filling with bone cement that crosses the midline and adheres to the endplate up and down is considered good dispersion; otherwise, it is considered poor dispersion. Chen et al. (32) demonstrated that the restoration of biomechanical balance depends on the distribution of cement. When choosing a unilateral pedicle approach, the bone cement is limited to one side of the vertebral body with poor dispersion, and there is a significant difference in strength between the two sides of the vertebral body. Compared to the strengthened side, the strength of the unreinforced side decreased significantly. The unevenly distributed bone cement causes the strength and stiffness of the vertebral body to show an asymmetric state in all regions. Local imbalance in biomechanics can exacerbate the overall biomechanical burden on the spine, leading to irreversible consequences, including other vertebral fractures (33, 34). Numerous studies and finite element analyses have shown that the uniform distribution of bone cement in the cancellous bone can reduce stress concentration, thereby further reducing the risk of recurrent fractures in adjacent and other vertebral bodies (35–37). Li et al. (12) found through a retrospective analysis of 329 surgical patients that patients with well-dispersed bone cement had a lower probability of recurrent fractures in adjacent vertebral bodies than patients with agglomerated and unevenly distributed bone cement. Theoretically, when bone cement is attached to the fractured vertebral endplate and distributed in a circular pattern, uneven biomechanical stress can be avoided, thereby reducing the risk of NVCF (38). In this study, we observed that good dispersion of bone cement was an important independent predictor of NVCF. Therefore, we believe that during the surgical process, if the surgeon chooses a unilateral pedicle approach, cement should be injected such that it spreads as far as possible to the opposite side and fits as close as possible to the upper and lower end plates. Compared to a unilateral approach, a bilateral pedicle approach can more easily achieve a uniform distribution of cement and reduce surgical difficulty (39), resulting in better surgical outcomes and a reduced risk of new vertebral fractures.

The vertebral endplate is the intermediate connection between the vertebral body and the intervertebral disc and is responsible for the smooth transmission of the mechanical load of the human body. It comprises two parts, including bone and cartilage endplates. The bony end plate shows mild depression. The cartilage endplate covers the bone endplate and exists for a lifetime, responsible for sharing the pressure of the intervertebral disc and exchanging nutrients with it (40). From the perspective of biomechanical research, the vertebral endplate bears 40–75% of the pressure of the vertebral body and directly participates in transmitting the pressure load from the intervertebral disc to the vertebral body (41). When the endplate fractures, the pressure it bears is uneven, leading to accelerated degeneration of the intervertebral disc (42). Studies have found that patients with endplate fractures who undergo cement surgery have an increased risk of cement leakage and may even experience fractures in other vertebral bodies (43). Based on these two points, when the bone cement seeps into the intervertebral disc through the fracture line of the endplate, it undergoes degeneration. At this time, the distance between the cement and adjacent vertebral endplate is shorter, and the stress on the adjacent endplate is more concentrated. Weak vibrations can amplify the load transmission of the cement on the adjacent endplate, thereby transmitting pressure to the adjacent or distal vertebral body and becoming a high-risk factor for postoperative recurrent fractures (1). In our study, endplate fracture was observed to be an independent predictor of refracture. We believe that it is necessary to control cement viscosity to prevent cement leakage during the surgical process, especially in patients with endplate fractures. Once leakage is detected during surgery, the cement injection should be stopped promptly. In addition, during balloon opening, the collapsed endplate should be restored completely to achieve complete anatomical reduction.

In recent years, an increasing number of scholars have focused on developing and validating predictive models for newly occurring vertebral fractures following vertebral augmentation procedures in osteoporotic compression fractures. Li et al. developed a predictive model incorporating factors such as the amount of bone cement injected, anti-osteoporosis treatment, cement leakage, cement dispersion, and endplate contact (12). This model demonstrated good discrimination, and the calibration curve indicated alignment between predicted values and actual outcomes, resulting in a higher clinical net benefit. In a model developed by Qian et al., predictors included bone mineral density (BMD), cement leakage, and cement morphology, with respective AUCs of 0.848 and 0.867 for the training and validation sets (16). Studies by Ma et al. identified female gender, cerebrovascular disease, fracture history, and cement intervertebral leakage as risk factors for NVCF (17). Zheng et al., in a population of postmenopausal individuals, found cement leakage, poor cement dispersion, and endplate fractures as independent predictors in their model for NVCF (44).

Other scholars have also conducted related research (18, 45). In existing prediction models, the types and quantities of predictive factors vary, but overall, they demonstrate high discrimination, calibration, and clinical net benefit. We carefully considered the reasons for this variation. Firstly, surgeons from different medical centers vary, and so do their surgical philosophies. For instance, some scholars believe that injecting more bone cement can yield better results, while others prioritize minimizing the risk of cement leakage. Secondly, the types and quantities of variables included by different scholars also vary, which could be another reason for differences in predictive factors. These studies include observed indicators comprising only general information and surgery-related factors, neglecting key laboratory test information in the pathogenesis of osteoporosis. Thirdly, due to their retrospective nature, data bias is inevitably present in these analyses.

In our study, we extracted 23 observed indicators, including results from tests for bone-specific alkaline phosphatase, vitamin D, and parathyroid hormone. This comprehensive inclusion of observed indicators aims to make our model more accurate and closer to the actual incidence rates. We endeavored to include as many relevant factors as possible in the database for analysis, encompassing all eligible patients treated by the same team over the past 7 years. Compared to other models, our developed model exhibits higher discrimination, calibration, and clinical net benefit while requiring fewer predictive factors. This enables clinicians to spend less time in practical settings making judgments based on predictive factors and achieve higher prediction success rates. This is beneficial for patient treatment and prognosis, aligning with the purpose of our research. Through our analysis, we identified three independent predictive factors: cement leakage, dispersion extent, and endplate fracture, and established a Nomogram. This Nomogram can be easily accessed through clinical imaging data. By using this model, spine surgeons can promptly and effectively assess the risk of NVCF, tailor surgical plans, postoperative treatment, and long-term management strategies for high-risk patients, thus avoiding waste of medical resources and alleviating the medical economic burden on patients and society.

Upon reviewing the literature, it was found that the majority of studies did not describe the specific methods for obtaining clinical data. In studies like that of TU et al., surgery-related preoperative and postoperative potential risk factors were extracted from medical records, surgical records, and questionnaires, but the extraction method was not mentioned. Radiological parameters postoperatively were manually assessed by spine surgeons (46). In our study, baseline characteristics and laboratory indicators were extracted using ETLCloud software, surgical factors were obtained by reviewing patient surgical records, and imaging information was assessed by spine surgeons using imaging system software. It can be imagined that the medical centers currently involved in the research have not yet achieved the automatic integration and presentation of patients’ general information, surgical records, imaging information, laboratory tests, etc., in real-time to clinicians, and feedback from clinicians cannot be received in real-time to adjust the model. Clinicians can only collect and integrate various information and analyze the model after treating a certain number of patients, correcting the model accordingly. This inevitably leads to lag in model adjustment. In summary, there have been no studies seen yet that systematically automatically extract all data, analyze, and provide feedback. This may be related to the level of informationization of various medical centers, which will be the direction of our next research. With further development and application of medical information technology and AI, the automatic identification, extraction, and real-time feedback of large amounts of clinical data will undoubtedly become a reality. The emergence of more clinical evidence and demographic data may make the model more stable and accurate.

This study has several limitations. First, the cases in this study were all from our hospital’s orthopedic surgical patients, lacking data comparison from multiple centers. External validation is needed with more patients from different countries and regions. Second, our study is a retrospective analysis, which entails a certain degree of selection bias. It is necessary to conduct prospective studies with large sample data jointly with other centers to verify the accuracy and applicability of the Nomogram. Third, the follow-up period in this study was 2 years, and longer follow-up is needed to validate the reliability of the model.

## Conclusion

Leakage and dispersion of bone cement, as well as the presence of fractured endplates, were the three independent predictive factors obtained in this study. We used these three factors to create a nomogram model and confirmed its discrimination, calibration, and clinical applicability through validation, which can help spinal surgeons better evaluate the risk of NVCF after PKP surgery and provide a basis for personalized clinical treatment plans and preventive measures, thereby reducing the incidence of NVCF, improving the quality of life of patients, and effectively avoiding the waste of medical resources.

## Data availability statement

The raw data supporting the conclusions of this article will be made available by the authors, without undue reservation.

## Ethics statement

The studies involving humans were approved by Ethics Committee of the Affiliated Hospital of Shandong University of Traditional Chinese Medicine. The studies were conducted in accordance with the local legislation and institutional requirements. Written informed consent for participation was not required from the participants or the participants' legal guardians/next of kin because the study will not have adverse effects on the health and rights of the subjects; the privacy and personal identity information of the subjects are protected. Written informed consent was not obtained from the individual(s) for the publication of any potentially identifiable images or data included in this article because this study will not have adverse effects on the health and rights of the subjects; the privacy and personal identity information of the subjects are protected.

## Author contributions

YG: Writing – original draft. JZ: Writing – original draft. KY: Data curation, Writing – review & editing. WW: Software, Writing – review & editing. GT: Investigation, Writing – review & editing, Methodology. JX: Formal analysis, Writing – review & editing. NL: Data curation, Supervision, Writing – review & editing. YC: Funding acquisition, Writing – review & editing.

## References

[1] Dai C Liang G Zhang Y Dong Y Zhou X. Risk factors of vertebral re-fracture after Pvp or Pkp for osteoporotic vertebral compression fractures, especially in eastern Asia: a systematic review and meta-analysis. J Orthop Surg Res. (2022) 17:161. doi: 10.1186/s13018-022-03038-z, PMID: 35279177 PMC8917756

[2] Buchbinder R Johnston RV Rischin KJ Homik J Jones CA Golmohammadi K. Percutaneous Vertebroplasty for osteoporotic vertebral compression fracture. Cochrane Database Syst Rev. (2018) 4:Cd006349. doi: 10.1002/14651858.CD006349.pub3, PMID: 29618171 PMC6494647

[3] Long Y Yi W Yang D. Advances in vertebral augmentation systems for osteoporotic vertebral compression fractures. Pain Res Manag. (2020) 2020:1–9. doi: 10.1155/2020/3947368 PMC7738798 33376566

[4] Sun HB Shan JL Tang H. Percutaneous vertebral augmentation for osteoporotic vertebral compression fractures will increase the number of subsequent fractures at adjacent vertebral levels: a systematic review and meta-analysis. Eur Rev Med Pharmacol Sci. (2021) 25:5176–88. doi: 10.26355/eurrev_202108_26531, PMID: 34486692

[5] Wang LJ Yang HL Shi YX Jiang WM Chen L. Pulmonary cement embolism associated with percutaneous vertebroplasty or kyphoplasty: a systematic review. Orthop Surg. (2012) 4:182–9. doi: 10.1111/j.1757-7861.2012.00193.x, PMID: 22927153 PMC6583132

[6] Nieuwenhuijse MJ Putter H van Erkel AR Dijkstra PDS. New vertebral fractures after percutaneous vertebroplasty for painful osteoporotic vertebral compression fractures: a clustered analysis and the relevance of intradiskal cement leakage. Radiology. (2013) 266:862–70. doi: 10.1148/radiol.12120751, PMID: 23204545

[7] Lindsay R Burge RT Strauss DM. One year outcomes and costs following a vertebral fracture. Osteoporos Int. (2005) 16:78–85. doi: 10.1007/s00198-004-1646-x, PMID: 15167988

[8] Cheng Y Cheng X Wu H. Risk factors of new vertebral compression fracture after percutaneous vertebroplasty or percutaneous kyphoplasty. Front Endocrinol (Lausanne). (2022) 13:964578. doi: 10.3389/fendo.2022.964578, PMID: 36120447 PMC9470857

[9] Seo DH Oh SH Yoon KW Ko JH Kim YJ Lee JY. Risk factors of new adjacent compression fracture after percutaneous vertebroplasty: effectiveness of bisphosphonate in osteoporotic or osteopenic elderly patients. Korean J Neurotrauma. (2014) 10:86–91. doi: 10.13004/kjnt.2014.10.2.86, PMID: 27169040 PMC4852619

[10] Yi X Lu H Tian F Wang Y Li C Liu H. Recompression in new levels after percutaneous vertebroplasty and kyphoplasty compared with conservative treatment. Arch Orthop Trauma Surg. (2014) 134:21–30. doi: 10.1007/s00402-013-1886-3, PMID: 24287674 PMC3889698

[11] Park JS Park YS. Survival analysis and risk factors of new vertebral fracture after vertebroplasty for osteoporotic vertebral compression fracture. Spine J. (2021) 21:1355–61. doi: 10.1016/j.spinee.2021.04.022, PMID: 33971326

[12] Li Q Long X Wang Y Fang X Guo D Lv J. Development and validation of a nomogram for predicting the probability of new vertebral compression fractures after vertebral augmentation of osteoporotic vertebral compression fractures. BMC Musculoskelet Disord. (2021) 22:957. doi: 10.1186/s12891-021-04845-x, PMID: 34784910 PMC8597210

[13] Lee BG Choi JH Kim DY Choi WR Lee SG Kang CN. Risk factors for newly developed osteoporotic vertebral compression fractures following treatment for osteoporotic vertebral compression fractures. Spine J. (2019) 19:301–5. doi: 10.1016/j.spinee.2018.06.347, PMID: 29959099

[14] Li YX Guo DQ Zhang SC Liang D Yuan K Mo GY. Risk factor analysis for re-collapse of cemented vertebrae after percutaneous vertebroplasty (Pvp) or percutaneous Kyphoplasty (Pkp). Int Orthop. (2018) 42:2131–9. doi: 10.1007/s00264-018-3838-6 29464371

[15] An Z Chen C Wang J Zhu Y Dong L Wei H. Logistic regression analysis on risk factors of augmented vertebra recompression after percutaneous vertebral augmentation. J Orthop Surg Res. (2021) 16:374. doi: 10.1186/s13018-021-02480-9, PMID: 34116683 PMC8194186

[16] Qian Y Hu X Li C Zhao J Zhu Y Yu Y. Development of a nomogram model for prediction of new adjacent vertebral compression fractures after vertebroplasty. BMC Surg. (2023) 23:197. doi: 10.1186/s12893-023-02068-6, PMID: 37430232 PMC10334643

[17] Ma Y Lu Q Wang X Wang Y Yuan F Chen H. Establishment and validation of a nomogram for predicting new fractures after Pkp treatment of for osteoporotic vertebral compression fractures in the elderly individuals. BMC Musculoskelet Disord. (2023) 24:728. doi: 10.1186/s12891-023-06801-3 37700293 PMC10496219

[18] Zhang A Fu H Wang J Chen Z Fan J. Establishing a nomogram to predict refracture after percutaneous kyphoplasty by logistic regression. Front Neuroinform. (2023) 17:1304248. doi: 10.3389/fninf.2023.1304248, PMID: 38187823 PMC10767997

[19] Bian F Bian G An Y Wang D Fang J. Establishment and validation of a nomogram for the risk of new vertebral compression fractures after percutaneous vertebroplasty in patients with osteoporotic vertebral compression fractures: a retrospective study. Geriatr Orthop Surg Rehabil. (2022) 13:215145932210986. doi: 10.1177/21514593221098620 PMC9073119 35529895

[20] Collins GS Reitsma JB Altman DG Moons KGM. Transparent reporting of a multivariable prediction model for individual prognosis or diagnosis (Tripod): the Tripod statement. BMJ. (2015) 350:G7594. doi: 10.1136/bmj.g7594 25569120

[21] Ballane G Cauley JA Luckey MM el-Hajj Fuleihan G. Worldwide prevalence and incidence of osteoporotic vertebral fractures. Osteoporos Int. (2017) 28:1531–42. doi: 10.1007/s00198-017-3909-3 28168409

[22] Voormolen MH Mali WP Lohle PN Fransen H Lampmann LE van der Graaf Y. Percutaneous vertebroplasty compared with optimal pain medication treatment: short-term clinical outcome of patients with subacute or chronic painful osteoporotic vertebral compression fractures. The VERTOS study. AJNR Am J Neuroradiol. (2007) 28:555–60. 17353335 PMC7977842

[23] Ko BS Cho KJ Park JW. Early adjacent vertebral fractures after balloon kyphoplasty for osteoporotic vertebral compression fractures. Asian Spine J. (2019) 13:210–5. doi: 10.31616/asj.2018.0224, PMID: 30481974 PMC6454291

[24] Lin WC Lee YC Lee CH Kuo YL Cheng YF Lui CC. Refractures in cemented vertebrae after percutaneous vertebroplasty: a retrospective analysis. Eur Spine J. (2008) 17:592–9. doi: 10.1007/s00586-007-0564-y, PMID: 18204942 PMC2295276

[25] Lin EP Ekholm S Hiwatashi A Westesson PL. Vertebroplasty: cement leakage into the disc increases the risk of new fracture of adjacent vertebral body. AJNR Am J Neuroradiol. (2004) 25:175–80. PMID: 14970015 PMC7974625

[26] Chen C Fan P Xie X Wang Y. Risk factors for cement leakage and adjacent vertebral fractures in kyphoplasty for osteoporotic vertebral fractures. Clin Spine Surg. (2020) 33:E251–5. doi: 10.1097/BSD.0000000000000928, PMID: 32011354

[27] Komemushi A Tanigawa N Kariya S Kojima H Shomura Y Komemushi S. Percutaneous vertebroplasty for osteoporotic compression fracture: multivariate study of predictors of new vertebral body fracture. Cardiovasc Intervent Radiol. (2006) 29:580–5. doi: 10.1007/s00270-005-0138-5, PMID: 16565797

[28] Sun YC Teng MM Yuan WS Luo CB Chang FC Lirng JF. Risk of post-vertebroplasty fracture in adjacent vertebral bodies appears correlated with the morphologic extent of bone cement. J Chin Med Assoc. (2011) 74:357–62. doi: 10.1016/j.jcma.2011.06.008, PMID: 21872816

[29] Rho YJ Choe WJ Chun YI. Risk factors predicting the new symptomatic vertebral compression fractures after percutaneous vertebroplasty or kyphoplasty. Eur Spine J. (2012) 21:905–11. doi: 10.1007/s00586-011-2099-5, PMID: 22160212 PMC3337901

[30] Ahn Y Lee JH Lee HY Lee SH Keem SH. Predictive factors for subsequent vertebral fracture after percutaneous vertebroplasty. J Neurosurg Spine. (2008) 9:129–36. doi: 10.3171/SPI/2008/9/8/129, PMID: 18764744

[31] Chen WJ Kao YH Yang SC Yu SW Tu YK Chung KC. Impact of cement leakage into disks on the development of adjacent vertebral compression fractures. J Spinal Disord Tech. (2010) 23:35–9. doi: 10.1097/BSD.0b013e3181981843, PMID: 20065868

[32] Chen B Li Y Xie D Yang XX Zheng ZM. Comparison of unipedicular and bipedicular kyphoplasty on the stiffness and biomechanical balance of compression fractured vertebrae. Eur Spine J. (2011) 20:1272–80. doi: 10.1007/s00586-011-1744-3, PMID: 21384203 PMC3175856

[33] Yu W Liang D Yao Z Qiu T Ye L Huang X. Risk factors for recollapse of the augmented vertebrae after percutaneous vertebroplasty for osteoporotic vertebral fractures with intravertebral vacuum cleft. Medicine (Baltimore). (2017) 96:E5675. doi: 10.1097/MD.0000000000005675, PMID: 28079799 PMC5266161

[34] Chevalier Y Pahr D Charlebois M Heini P Schneider E Zysset P. Cement distribution, volume, and compliance in vertebroplasty: some answers from an anatomy-based nonlinear finite element study. Spine (Phila Pa 1976). (2008) 33:1722–30. doi: 10.1097/BRS.0b013e31817c750b, PMID: 18628704

[35] Chen JB Xiao YP Chen D Chang JZ Li T. Clinical observation of two bone cement distribution modes of percutaneous vertebroplasty in the treatment of thoracolumbar Kümmell's disease. J Orthop Surg Res. (2020) 15:250. doi: 10.1186/s13018-020-01774-8, PMID: 32646461 PMC7346457

[36] He S Zhang Y Lv N Wang S Wang Y Wu S. The effect of bone cement distribution on clinical efficacy after percutaneous kyphoplasty for osteoporotic vertebral compression fractures. Medicine (Baltimore). (2019) 98:E18217. doi: 10.1097/MD.0000000000018217, PMID: 31852080 PMC6922577

[37] Yao G Shen YX Li M Cai B. Biomechanical effects of different bone cement diffusion patterns after vertebroplasty:finite element analysis. Zhongguo Gu Shang. (2021) 34:732–7. doi: 10.12200/j.issn.1003-0034.2021.08.008, PMID: 34423616

[38] Baek SW Kim C Chang H. The relationship between the spinopelvic balance and the incidence of adjacent vertebral fractures following percutaneous vertebroplasty. Osteoporos Int. (2015) 26:1507–13. doi: 10.1007/s00198-014-3021-x 25619632

[39] Zhang Y Chen X Ji J Xu Z Sun H Dong L. Comparison of unilateral and bilateral percutaneous kyphoplasty for bone cement distribution and clinical efficacy: an analysis using three-dimensional computed tomography images. Pain Physician. (2022) 25:E805–13. PMID: 36122263

[40] Moore RJ. The vertebral endplate: disc degeneration, disc regeneration. Eur Spine J. (2006) 15:333–7. doi: 10.1007/s00586-006-0170-4 PMC2335377 16816945

[41] Moura DL Gabriel JP. Expandable intravertebral implants: a narrative review on the concept, biomechanics, and outcomes in traumatology. Cureus. (2021) 13:E17795. doi: 10.7759/cureus.17795, PMID: 34660005 PMC8496495

[42] Su Y Ren D Chen Y Geng L Yao S Wu H. Effect of endplate reduction on endplate healing morphology and intervertebral disc degeneration in patients with thoracolumbar vertebral fracture. Eur Spine J. (2023) 32:55–67. doi: 10.1007/s00586-022-07215-w, PMID: 35435517

[43] Zhao Z Deng L Hua X Liu H Zhang H Jia X. A retrospective study on the efficacy and safety of bone cement in the treatment of endplate fractures. Front Surg. (2022) 9:999406. doi: 10.3389/fsurg.2022.999406, PMID: 36277290 PMC9585934

[44] Zheng J Gao Y Yu W Yu N Jia Z Hao Y. Development and validation of a nomogram for predicting new vertebral compression fractures after percutaneous kyphoplasty in postmenopausal patients. J Orthop Surg Res. (2023) 18:914. doi: 10.1186/s13018-023-04400-5, PMID: 38037128 PMC10688465

[45] Mao Y Wu W Zhang J Ye Z. Prediction model of adjacent vertebral compression fractures after percutaneous kyphoplasty: a retrospective study. BMJ Open. (2023) 13:e064825. doi: 10.1136/bmjopen-2022-064825, PMID: 37258076 PMC10255151

[46] Tu W Niu Y Su P Liu D Lin F Sun Y. Establishment of a risk prediction model for residual low back pain in thoracolumbar osteoporotic vertebral compression fractures after percutaneous kyphoplasty. J Orthop Surg Res. (2024) 19:41. doi: 10.1186/s13018-024-04528-y, PMID: 38184651 PMC10771681

