# Supplementary material for: Construction of a nomogram to predict the probability of new vertebral compression fractures after vertebral augmentation of osteoporotic vertebral compression fractures: a retrospective study
Source: Front Med (Lausanne). 2024 Apr 23;11:1369984. doi: 10.3389/fmed.2024.1369984 (PMC11074446; doi:10.3389/fmed.2024.1369984)
Supplement: Supplementary file 1 [file Data_Sheet_1.PDF]

File1:Comparison of different prediction models

Model	Observaion indicators	Statistical methods	Medical center	Number of surgical teams	Amount of bone cement (training set)	Amount of bone cement (testing set)	Follow up time	Sample size	Training and test data ratio	AUC (training set)	AUC (testing set)	Predictors
Li (2021)	(1) General information: gender, population, age, body mass index (BMI), and fractured vertebral body segments.<br>(2) Surgical factors: surgical method, average bone cement dosage, bone cement leakage, bone cement dispersion, contact between bone cement and endplate, regular anti-osteoporosis treatment.<br>(3)Comorbidities: history of diabetes, hypertension, and fractures.<br>(Total 20 variables)	Categorical variables were expressed as rates, and the chi-square test was used for comparison between groups.Continuous variables are presented as mean values ± standard deviation, and independent samples t-test or analysis of variance (ANOVA) was used for comparison between groups. The texture feature selection using least absolute shrinkage and selection operator (LASSO) regression model is used for data dimensionality reduction and feature selection.	People's Hospital of Ningxia Hui Autonomous Region	One	4.43 ± 0.88 ml	4.02 ± 1.13 ml	Two years	562	71.7% to 28.3%	0.882	0.869	Age, Bone cement dosage, Anti-osteoporosis treatment, Bone cement leakage, Bone cement dispersion, Contact with endplate

Qian (2023)	(1) Systemic related factors: sex, age, body mass index (BMI), BMD, fracture segment, number of fractured vertebrae, history of diabetes, and history ofhypertension.<br>(2) Surgical related factors: surgical method, dosage of bone cement, shape of bone cement, dispersion distribution grade of bone cement,leakage of bone cement, anterior vertebral height(AVH) restoration, kyphotic anglerestoration, and Cobb angle restoration. (Total 16 variables)	SPSS 22.0 version was used for statistical analysis of related risk factors. Measurement data are represented asmedian, and categorical data are presented as percentages. Variables were tested for normality using the Shapiro–Wilk test. For data with normal distribution, differences between groups were analyzed using a t-test. The Mann–Whitney U test was used to compare data that were not normally distributed. A Chi-square test or Fisher exact test was used for enumeration data. Wilcoxon rank sum test was used for ranked data. $P < 0.05$ was considered statistically significant. R version 4.0.0 was used for nomogram model establishment and verification. Least absolute shrinkage and selection operator (LASSO regression) and logistic regression was used to screen the independent risk factors, and R was used to establish the risk prediction model.	Tongji Hospital, Tongji University School of Medicine	One	Unstatistical mean and standard deviation	Unstatistical mean and standard deviation	The median follow-up time of training cohort was 33.39(28.88 ~ 38.09)months and the median follow-up time of validation cohort was 17.77(15.04 ~ 20.50)months	509	65.2% to34.8%	0.848	0.867	BMD, leakage of bone cement, shape of bone cement

Zhang (2023)	age, sex, diabetes, hypertension history, body mass index, BMD, the vertebral segment of the fracture, the average volume of bone cement used, bone cement leakage, bone cement dispersion, bone cement distribution ,contact of bone cement with the endplate, osteoporosis treatment,spinal curvature, preoperative and postoperative Cobb angle, preoperative and postoperative anterior vertebral height (AVH) (Total 19 variables)	Data analysis and graphical representation utilized IBM SPSS Statistics for Windows, version 26 and R 4.22 software. Continuous variables were expressed as means ± standard deviations and compared using independent samples t-tests. Categorical variables were presented as percentages and compared using chi-square tests. Lasso regression aided in the selection of risk factors. The parameters selected by Lasso regression informed the construction of logistic regression and RF predictive models.	Zhejiang Provincial People's Hospital	One	4.90 ± 1.25 ml in Control group and 4.82 ± 1.16 in NVCF group	4.90 ± 1.25 ml in Control group and 4.82 ± 1.16 in NVCF group	Not involved	349	70% to 30%	0.868	0.786	BMD, Augmentation segment, shape D, Pre-op AVH, AVHRR

Ma (2023)	(1) General information: sex, age, body mass index (BMI), chronic diseases (hypertension, diabetes, respiratory diseases, heart diseases and cerebrovascular diseases), fracture history, time of injury, time from admission to surgery, location of fractured vertebrae, number of fractured vertebrae, and type of fracture.<br>(2) Surgical factors: surgical approach (unilateral or bilateral), new vertebral fracture, type of cement leakage (paravertebral leakage, intervertebral leakage, spinal leakage), cement distribution,cement-to-endplate contact, vertebral height recovery rate, and postoperative Cobb angle.<br>(Total 25 variables)	Continuous variables were expressed as the mean ± standard deviation, and categorical variables were expressed as ratios. Univariate and multivariate logistic regression analyses were used to filter the variables in the dataset, and variables with P < 0.05 were included in the model. The "rms" package in R-Studio was used to build the nomogram.	Xuzhou Medical University	One	Not involved	Not involved	28.92 ± 4.21 months	529	75% to 25%	0.795	0.861	Female sex, Cerebrovascular disease, Fracture history, Bone cement intervertebral leakage
Bian (2022)	general characteristics (e.g., age, sex, BMI), radiological characteristics (e.g., Hu value,kyphotic angle,the volume of bone cement,leakage of cement, fracture level), and previous history (e.g., smoking, alcohol, fracture history, and bisphosphonate therapy).<br>(Total 15 variables)	SPSS 23 and R software were used for statistics and analysis. The Continuous variables were expressed as the mean ± standard deviation. The categorical variables were statistically analysed using the chi-square test and reported as percentages.	Daqing Oilfield General Hospital, Chengde Medical University Affiliated Hospital	Two	4.70 ± .80 (ml)	3.12 ± .77(ml)	All patients was greater than or equal to 2 years.	562	70% to 30%	0.85	0.88	Age, cement leakage, TL junction, Hu value
