# Supplementary figures and images for: Construction of a nomogram to predict the probability of new vertebral compression fractures after vertebral augmentation of osteoporotic vertebral compression fractures: a retrospective study
Source: Front Med (Lausanne). 2024 Apr 23;11:1369984. doi: 10.3389/fmed.2024.1369984 (PMC11074446; doi:10.3389/fmed.2024.1369984)

**Nomogram operation flowchart**

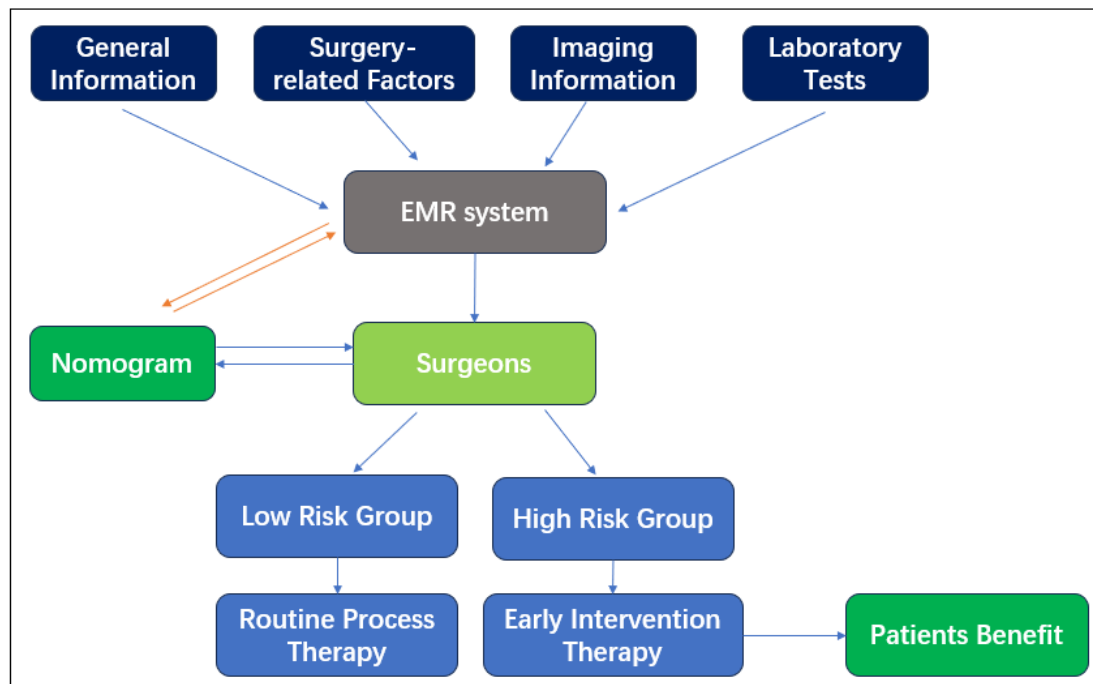

Supplement: Supplementary file 2 [file Data_Sheet_2.PDF]
